# Gas-Particle Distribution of D_5_ Oxidation Products in New York City during Summertime

**DOI:** 10.1021/acsestair.5c00193

**Published:** 2025-10-16

**Authors:** Josie K. Welker, Jeewani N. Meepage, Charles O. Stanier, Elizabeth A. Stone

**Affiliations:** † 4083 University of Iowa, Department of Chemistry, Iowa City, Iowa 52242, United States; ‡ University of Iowa, Department of Chemical and Biochemical Engineering, Iowa City, Iowa 52242, United States

**Keywords:** 1-hydroxynonamethylcyclopentasiloxane, secondary organic aerosol, gas-particle distribution, positive artifact, SOA tracer, personal care products

## Abstract

Personal care products can release decamethylcyclopentasiloxane (D_5_) to the atmosphere, where it oxidizes to form 1-hydroxynonamethylcyclopentasiloxane (D_4_TOH). This oxidation product subsequently can partition to the particle-phase to form secondary organic aerosol (SOA). The gas-particle distribution of D_4_TOH has been studied in the laboratory but has yet to be established in ambient air. This study examines the gas-particle distribution of D_4_TOH and related oxidation products in New York City during the summertime of 2022 using medium volume air samplers, solvent extraction, and gas and liquid chromatography mass spectrometry methods. Positive sampling artifacts constituted the majority of D_4_TOH observed on quartz fiber filters (54–100%, averaging 86%, *n* = 12), indicating the high potential for particle-phase D_4_TOH to be overestimated. After artifact correction, D_4_TOH was observed in fine particles in 5 of the 12 sampling periods, with its particle-phase fraction averaging 13%. Because D_4_TOH is predominantly in the gas phase, it makes a minor contribution to D_5_-derived SOA during summertime. Further oxidation products of D_5_, including di, and tetrasiloxanols are predominantly in the particle-phase (>77%, *n* = 4) during summertime and have relatively small positive artifacts. These polysiloxanols provide evidence of D_5_-derived SOA in the urban aerosols and are more suitable tracers for D_5_-derived SOA than D_4_TOH in summertime because of their higher particle-phase fractions.

## Graphical abstract

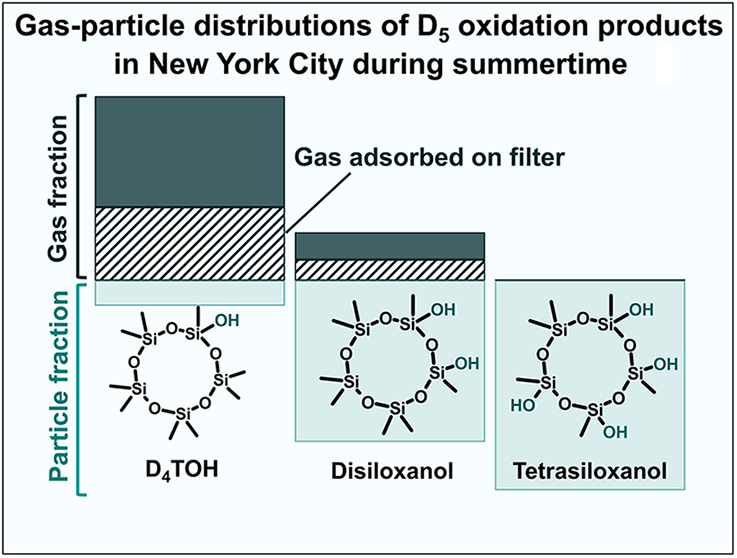

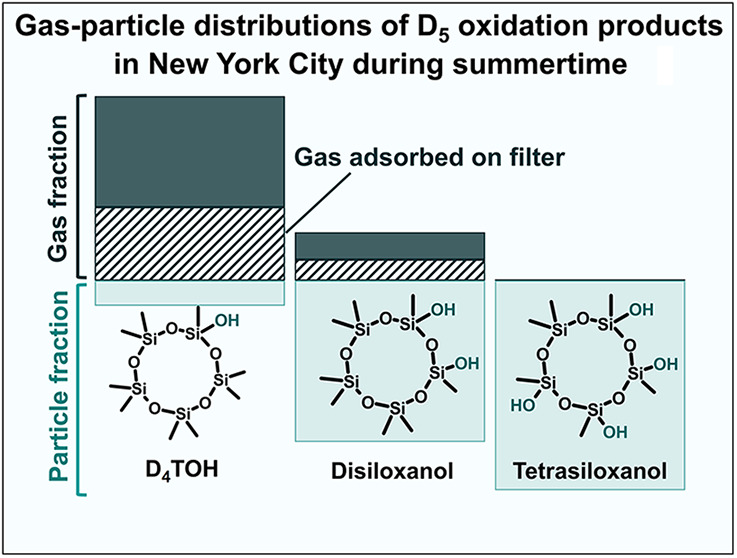

## Introduction

Personal care products, such as deodorants, lotions, and perfumes, often contain decamethylcyclopentasiloxane, or D_5_.^1,2^ Due to its high vapor pressure, D_5_ evaporates quickly after product application.^3^ Peak atmospheric concentrations of D_5_ have been observed in and downwind of urban areas, agreeing with modeled emissions based on population density.^4,5^ In locations with industrial use of siloxanes or silicone manufacturing, D_5_ enhancements have been observed as well.^6,7^ D_5_ has an atmospheric lifetime of 4–10 days, enabling long-range intrahemispheric transport.^8–14^ D_5_ has been observed in remote environments, like Antarctica, and is recognized as being environmentally persistent.^14–21^ In the atmosphere, D_5_ can react with hydroxyl radicals, and to a lesser extent chlorine radicals, to create several oxidation products.^9,22–29^ The major oxidation product of D_5_ has been identified in prior studies as 1-hydroxynonamethylcyclopentasiloxane, or D_4_TOH.^25,27,28^ Additional D_5_ oxidation products include a series of siloxanols, functionalized cyclic siloxanes, dimers, and ring-opened species.^27,28,30^ D_5_ oxidation products have been observed in urban and traffic aerosols, although their ambient concentrations and contributions to ambient fine particulate matter (PM_2.5_) are yet to be established.^8,31,32^ To better understand the impacts of D_5_ on atmospheric secondary organic aerosol (SOA) concentrations, this study examines the gas-particle distribution of D_4_TOH and related products.

As D_5_ undergoes atmospheric oxidation, compounds with oxygenated functional groups, like siloxanols, are formed.^5,15,25,27–30,33^ These oxidation products can partition to the particle-phase more readily than D_5_ because of their higher water solubility and lower vapor pressures.^5,15,25,27,30,33^ Gas-particle distributions are typically represented by the partitioning coefficient, *K*_p_, which divides the particle and gas-phase concentration ratio (*C*_p_/*C*_g_) by the suspended particulate concentration (*C*_TSP_) and accounts for the linear increase in *C*_p_/*C*_g_ with increasing *C*_TSP_.^26^ Latimer et al.^26^ reported that D_4_TOH was almost entirely in the gas-phase in the presence of wood and coal particles, while it partitioned almost entirely to the particle-phase under cooler temperatures (0 °C) with diesel and mineral dust particles. Chandramouli and Kamens^25^ show that the partitioning of D_4_TOH to mineral dust was highly efficient, to the extent where D_4_TOH was immeasurable in the gas-phase. This partitioning is proposed to occur via adsorption, due to energetically favorable interactions between D_4_TOH and silicon dioxide.^25^ The effects of humidity on partitioning varied with aerosol type, with D_4_TOH partitioning to dust particles decreasing with increasing humidity, likely due to the competition of D_4_TOH with water for sorption sites, while its partitioning to wood and coal particles increased with increasing humidity.^26^ To the best of our knowledge, prior studies have yet to determine the gas-particle distribution or *K*_p_ of D_4_TOH in ambient aerosol. Establishing ambient D_4_TOH gas-particle distributions is important because they apply to a complex, real-world environment, under atmospherically relevant concentrations.

Accurate determination of gas-particle distributions requires adequate evaluation and treatment of sampling artifacts that can artificially bias gas or particle concentrations.^34^ Positive sampling artifacts can artificially increase particle-phase concentrations when gas-phase compounds sorb to the particle-phase substrate, which can be the case for quartz fiber filters (QFF) that have large surface areas.^35^ Positive sampling artifacts can be addressed through a variety of methods, including filter–filter-denuder, or filter-backup filter sampling setups.^35^ In their chamber experiments of D_5_ and D_4_TOH, Chandramouli and Kamens^25^ used a filter–filter-denuder setup to account for gas adsorption on filters, assuming that front and back-up filters quickly reached equilibrium due to high gas-phase concentrations. Because the positive artifact saturates as available adsorption sites are filled while deposited particles generally accumulate over time, bias due to not correcting for positive artifact is most severe with lightly loaded filters; this may occur due to low ambient loadings, short sampling times, or both.^36^

This work examines the gas-particle distribution and sampling artifacts of D_5_ oxidation products during the New York City metropolitan Measurements of Emissions and TransformationS (NYC-METS) field study from July to August 2022. Co-located measurements by Hass-Mitchell et al.^37^ with an aerosol chemical speciation monitor previously demonstrated that PM_1_ was predominantly organic aerosol (averaging 80–83%), with substantial influences from SOA production. The objectives of this study are to establish gas-particle distributions of D_5_ oxidation products, particularly D_4_TOH and further D_5_ oxidation products (i.e., di and tetrasiloxanols), while correcting for positive sampling artifacts. For correction of gas sorption to QFF, front filters (QFF_f_) used to collect particle-phase samples, were paired with backup QFF (QFF_b_) that captured adsorbed gases. Gas chromatography coupled with mass spectrometry (GC-MS) was applied to measure D_4_TOH, while D_4_TOH and other D_5_ oxidation products were measured via liquid chromatography mass spectrometry (UPLC-MS/MS). Gas-particle distributions of oxidized D_5_ products are compared to polycyclic aromatic hydrocarbons (PAH), the Pankow absorptive partitioning model,^38^ and the Junge-Pankow adsorption model^39^ to gain insight into the molecular properties and processes that affect their ambient phase distributions. Understanding the gas-particle distribution of D_4_TOH and other D_5_ oxidation products advances our understanding of its ability to contribute to SOA in a densely populated urban airshed.

## Materials and Methods

### Field Measurements in New York City

Field measurements were conducted at the Advanced Science and Research Center (ASRC, 40°48′55.5″N, 73°57′01.5″W) at the City College of New York in Harlem, Manhattan, New York City, New York, USA during the NYC-METS field campaign.^8,37^ The field site is located near downtown Manhattan and is a heavily populated urban area, situated between the Harlem River and the Hudson River on either side. All personnel working near the sampling site agreed to refrain from using D_5_-containing personal care products during the periods in which they were present in the active sampling sites from July 6–August 6, 2022, to help prevent potential contamination. Co-located relative humidity data was also collected using a Vaisala weather station for the PUF period sampling dates.^37^

### PM_2.5_ and Gas Sample Collection

PM_2.5_ and gas samples were collected from July 25–August 3, 2022 using two medium volume samplers (URG-3000 ABC). Samplers were secured to the rooftop of the ASRC with sampler inlets at 88 m above sea level.^37^ The URG samplers operated at a typical flow rate of 90 L min^–1^, with air flow rate measured before and after sample collection using a rotameter (Gilmont Instruments). Samples were collected every 12 h (“N” after the date for nighttime samples and “D” after the date for daytime samples). One field blank was collected for every five samples following identical handling protocols as samples, with the exception of active air flow.

PM_2.5_ and gas samples were collected onto QFF and PUF substrates, respectively. Prior to sample collection, QFF 90 mm quartz fiber filters (QFF; Tissuquartz, Pall Life Sciences, East Hills, New York) were precleaned by heating to 550 °C for 18 h to remove any organic matter before sample collection. Polyurethane foam filters (PUF, URG) were precleaned with ultrapure water, acetonitrile (Fisher Scientific Company, >99.9%), and acetone (Fisher Scientific Company, >99.9%) by sonication (Branson 5510, 137 W) and repeated compressions.

The two URG air samplers were configured as shown in Figure 1 from July 24–July 31 and August 2–3, 2022 for assessment of sampling artifacts and gas-particle distribution of organic carbon (OC) and organic compounds. Sampler A collected PM_2.5_ on the front filter and semivolatile gases on the PUF. For assessment of sampling artifacts due to sorption of gases on QFF, sampler B was configured with a QFF_f_ and QFF_b_. The sampling configuration did not include QFF_b_ from the nighttime sample of July 31 to the daytime of August 2, 2022. After collection, QFF were stored in aluminum-foil lined Petri dishes sealed with Teflon tape. PUFs were transferred to prebaked wide-mouth amber jars with Teflon-lined caps and sealed with Teflon tape. Sampled QFF and PUFs were placed in zipper bags and transported to a laboratory for storage at −20 °C.

**Figure 1:**
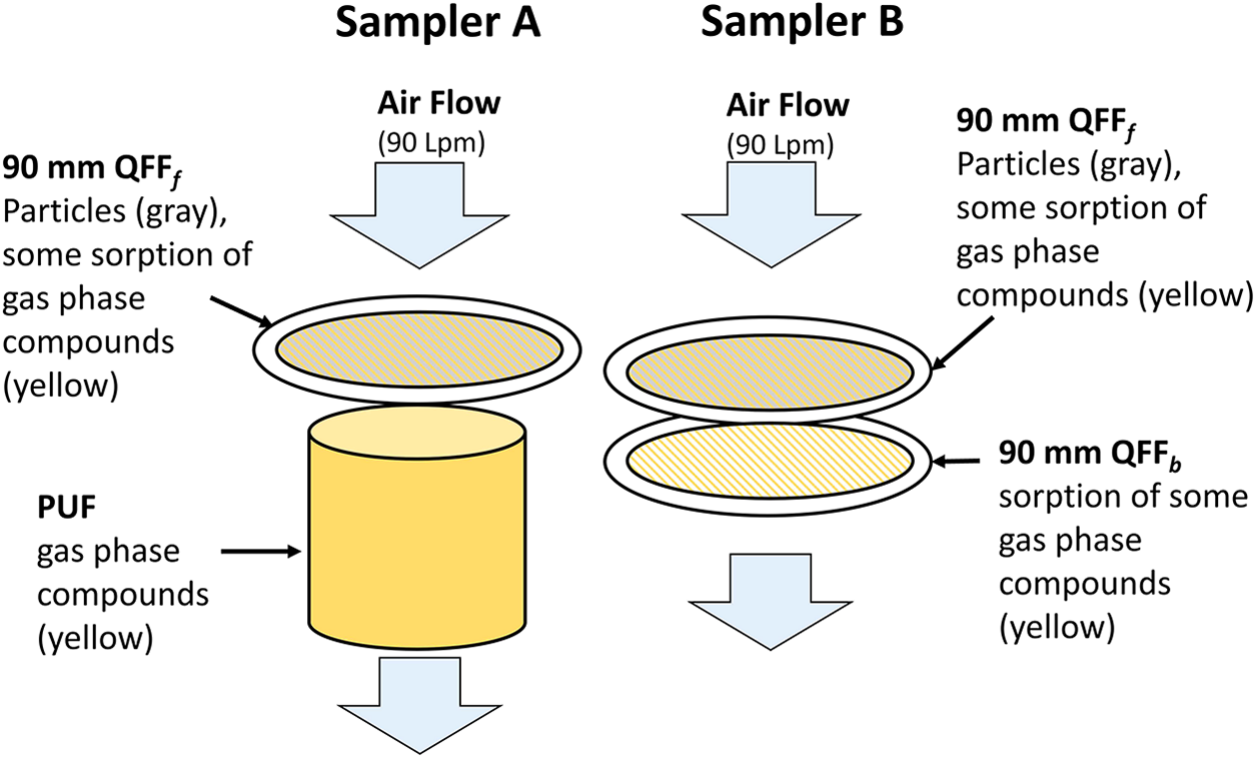
Schematic of gas- and particle-phase sampling setup where particles are indicated by the color gray and gas-phase compounds are indicated by yellow.

### Organic and Elemental Carbon Analysis

OC and elemental carbon (EC) were measured by thermal-optical analysis (Sunset Laboratory Inc.) on a 1 cm^2^ filter punch.^40^ OC and EC analysis was applied to QFF_f_, QFF_b_, and field blanks from sampler B. EC was not detected in field blanks. Field blank OC concentrations were averaged (*x̅*: 0.31 μg cm^–2^, σ: 0.12 μg cm^–2^) and subtracted from each sample concentration. Concentrations were then converted to μg m^–3^ using sampled air volume (m^3^) and sampled area (cm^2^) for each sampling duration. Analytical uncertainties were propagated from the standard deviation of the field blanks and a fraction of the measurement, which for OC was 20% and for EC was 5% of EC plus 5% of pyrolyzed carbon.

### Sample Extraction and Analysis by GC-MS

For determination of PAHs and D_4_TOH by GC-MS, QFF and PUF samples underwent solvent-extraction. The extraction procedure followed an established protocol previously used to determine the gas-particle partitioning of PAHs^41^ and to detect D_4_TOH ambient air.^31^ A fraction of the filters from sampler B were extracted, equivalent to 45.3 cm^2^ of the QFF_f_ and 37.7 cm^2^ for QFF_b_ from a sampled filter area of 50.3 cm^2^. The subsample of filter was placed in a jar and spiked with isotopically labeled internal standards, acenaphthene-D_10_, pyrene-D_10_, benz­[*a*]­anthracene-D_12_, coronene-D_12_, and pentadecane-D_32_. The filters were then extracted by sonication (Branson 5510, 137 W) in three 10 mL aliquots of acetonitrile (Fisher Scientific Company, >99.9%) for 10 min at 60 sonics per minute. Previous studies show that sonication can create radicals that react with compounds in the PM samples, which can cause positive or negative artifacts in the extraction process;^42,43^ however, such artifacts introduced by sonication cannot be assessed due to lack of authentic standards. Extracts were combined into a round-bottom flask and evaporated via rotary evaporator (Heizbad Hei-VAP, Heidolph) to 1 mL at 30 °C, 120 rpm, and 200 mbar. The 1 mL extract was filtered through a 0.25 μm PFTE filter (Whatman, GE Health Care Life Sciences) and evaporated to a final volume of 40 μL under a gentle stream of high-purity nitrogen (99.999%, Linde) at 30 °C (Reacti-Vap I, Thermo Scientific). PUF samples were initially extracted via compression in 3 aliquots of 150 mL acetonitrile (Fisher Scientific Company, >99.9%).^41^ Subsequently, these aliquots were combined in a round-bottom flask and were reduced in volume following the same procedure as the QFF. Samples were stored frozen at −20 °C until analysis. Spike recovery data, which shows an acceptable range of recoveries for PAHs in both QFF and PUF materials is provided in the Supporting Information (Figure S1).

Sample extracts were analyzed by GC-MS (Agilent 7890A GC, coupled with 5975C MS) equipped with a DB-5 column (30 m × 0.25 mm × 0.25 μm; Agilent; Santa Clara, CA). Other GC-MS conditions were as follows: helium carrier gas (>99.999%, Linde); GC inlet temperature of 300 °C; 2 μL injection volume; GC oven temperature initially held at 65 °C for 10 min, then ramped at 10 °C min^–1^ to a final temperature of 300 °C, and held for 26.5 min; and MS acquisition under electron ionization (EI) at 70 eV, mass range of 50–550 Da, source temperature of 230 °C, and quadrupole temperature of 150 °C.^31,44,45^

PAH were quantified by internal standard-normalized five-point linear calibration curves (*R* ^2^ ≥ 0.995). Because no authentic standard of D_4_TOH was commercially available at the time of this study, its concentrations were semiquantified through isotopic dilution using pentadecane-D_32_ as the internal and surrogate standard. Pentadecane-D_32_ has a similar volatility to D_4_TOH as indicated by its GC retention time^31^ and can account for volatile losses during sample preparation,^46^ as well as injection and sample volume variations. The use of a surrogate standard in semiquantification may introduce bias in absolute quantification when detector response of the analyte varies from that of the selected internal standard. While the extent of such bias is unknown, the use of semiquantification does not affect the determination of the gas-particle distribution because the detector response factor proportionally affects gas and particle-phase concentrations and is factored out. The response factor likewise cancels in determining artifact fractions, so that they can also be accurately determined in the absence of an authentic standard.

### Sample Extraction and Analysis for UPLC-MS/MS

UPLC-MS/MS analysis was performed on extracts of both gas- and particle-phase samples on a Q-Exactive Quadrupole Orbitrap mass spectrometer (Thermo Scientific) operated in negative mode with a heated electrospray ionization source, following conditions described by Meepage et al.^8^ Subsamples of QFF_f_ from sampler A and QFF_b_ from sampler B, both with equivalent areas of 11.6 cm^2^, were extracted twice sequentially by sonication (30 min each, 60 sonics min^–1^, Branson 5510, 137 W) with acetonitrile (Fisher Scientific Company, >99.9%) and ultrapure water (95:5, 10 mL). The combined extracts were filtered through polypropylene membrane syringe filters (0.45 μm followed by 0.20 μm pore size, Puradisc, Whatman), and the volumes were reduced to 500 μL under a stream of ultrahigh purity nitrogen gas (99.999%, Linde) (≤5 psi) at 50 °C using a Turbovap LV evaporation system (Caliper Life Sciences). The extracts were transferred to LC vials (1.5 mL, Agilent) and evaporated to near dryness (50 μL) under a very light stream of ultrahigh purity nitrogen gas (99.999%, Linde) at 50 °C using a microscale nitrogen evaporation system (ReactiTherm III TS 18824 and Reacti-Vap I 18825, Thermo Scientific). They were then reconstituted in 90 μL of acetonitrile (Fisher Scientific Company, >99.9%): ultrapure water (95:5). Finally, 10 μL of 1000 μg L^–1^ D_5_-phenol (98% D, Sigma-Aldrich) was added as an isotopically labeled internal standard to bring the final volume to 100 μL. The same PUF extracts were used for GC-MS and UPLC-MS/MS analysis, with the extraction for PUFs described previously.

Due to the unavailability of authentic siloxanol standards, semiquantitative analysis was applied in which tris­(tert-butoxy)­silanol (Sigma-Aldrich) was used as a surrogate standard. Calibration curves were prepared at concentrations ranging 4–3800 nmol L^–1^ (*r* ^2^ ≥ 0.995). The lowest quantifiable peak using the UPLC-MS/MS method corresponded to surrogate standard concentrations equivalent to 8 pg m^–3^ for the QFF and 14 pg m^–3^ for PUF. Data were acquired using Xcalibur 4.2 software (Thermo Scientific), and semiquantification was conducted with TraceFinder v4.0.

### Field Blank and Artifact Correction

All measurements were field blank subtracted using the average field blank concentration for each compound. For artifact correction, compound concentrations were calculated by subtracting the observed QFF_b_ concentration from that of QFF_f_. This calculation assumes that gas adsorption on the front and backup filters is equivalent, which is consistent with gas adsorption being dominated by the substrate and not the collected PM. The sample volume (65 m^3^) and face velocity (29 cm s^–1^) used in this study are assumed to reach equilibrium between front and back filters in the 12 h sampling period, based on prior studies by Subramanian et al. 2004 ^36^ that reported good agreement between QFF backup subtraction and a denuder-based method for OC samples collected with a lower sample volume of 24 m^3^ and the same face velocity (29 cm s^–1^). The artifact correction calculation allowed for differentiation of PM_2.5_ and adsorbed gases, and the extent of the positive artifact on QFF_f_ was calculated using eq 1.

1 $$p\mathrm{ositive artifact}\;\left(\%\right)=\left({\mathrm{QFF}}_{b}/{\mathrm{QFF}}_{f}\right)\times 100\%$$

### Gas-Particle Distributions

For the assessment of gas-particle distributions of D_5_ oxidation products and PAHs, artifact-corrected PM_2.5_ and gas concentrations were used. The particle phase fraction (*F*_p_) was calculated as the ratio of the artifact-corrected particle concentration to the sum of gas and particle concentrations, using eq 2.^41^

2 $$F_{p}\;\left(\%\right)=\frac{{[\mathrm{QFF}}_{f}-{\mathrm{QFF}}_{b}]}{\left[\mathrm{PUF}\right]+\left[{\mathrm{QFF}}_{f}\right]}\times 100$$

### PM_2.5_, TSP, and *K*_p_ Estimations

For the calculation of *K*_p_, (eq 3) the ratio of the particle and gas phase concentrations (*C*_p_/*C*_g_) were divided by our best estimate of the TSP concentration (*C*_TSP_).

3 $$K_{p}=\frac{C_{p}}{C_{g}C_{\mathrm{TSP}}}$$

Because *C*_TSP_ was not measured, it was estimated as from our measurements of PM_2.5_ OC, the OC fraction of PM_2.5_ (37%)^47^ and the PM_2.5_ to PM_10_ ratio (1.8) assuming that PM_10_ concentrations are approximately equal to TSP.^47^

### Absorptive and Adsorptive Partitioning Model

*K*_p_ and *F*_p_ for the PAH and for D_4_TOH were predicted using the Pankow absorptive and Junge-Pankow adsorptive partitioning model, with details of equations and variables in the Supporting Information (eq S1).^38^ The adsorptive partitioning in this model is based on Langmuir adsorption theory, which assumes monolayer adsorption.

## Results and Discussion

### Organic Carbon Values and Positive Artifacts

PM_2.5_ OC concentrations from July 25–August 3, 2022 ranged from 1.8–5.6 μg m^–3^ (Figure S2). Daytime (D) OC concentrations averaged 3.6 μg m^–3^, while nighttime (N) concentrations averaged 3.4 μg m^–3^. EC concentrations ranged from 0.19–0.56 μg m^–3^ with a daytime average of 0.28 μg m^–3^ and a nighttime average of 0.36 μg m^–3^. OC/EC ranged from 9.8–22.1 and the higher average daytime OC/EC ratio (11.9) compared to average nighttime (9.0) is consistent with photochemical SOA formation during the daytime.

OC positive artifacts, attributed to gas adsorption on QFF, ranged from 11%–22% with an average of 16% from July 24 (D) to August 3 (N), excluding dates where there was no collection of QFF_*b*_ (Figure 2). The magnitude of sampling artifacts fell within the lower end of the range observed in prior studies in North America (17–44%) using a backup QFF.^36,48^ EC, which is expected to be entirely in the particle phase, did not have any detectable positive artifacts, indicating that there was no detectable breakthrough of PM from QFF_f_ to QFF_b_.

**Figure 2:**
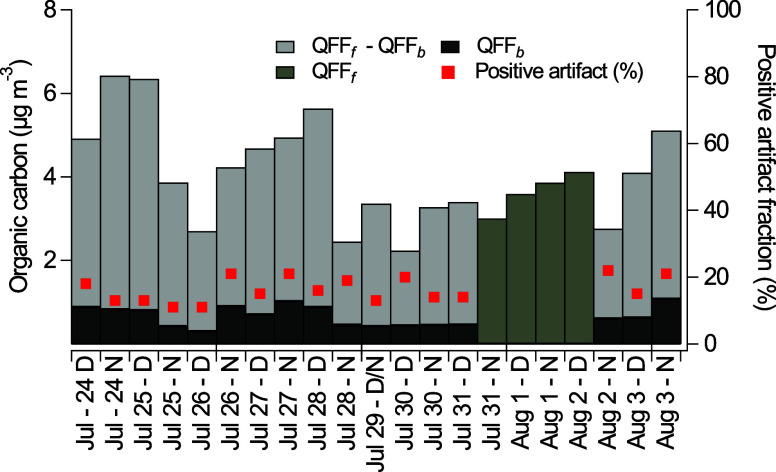
Organic carbon concentrations in NYC measured on front (QFF_f_) and back (QFF_b_) filters in each 12 h sampling period, with “N” and “D” denoting nighttime and daytime samples, respectively. The percentage of the QFF positive artifact, determined by eq 1, is evaluated relative to the total OC measured and is indicated by red squares. Days without QFF_b_ collection are denoted by dark gray bars.

### D_4_TOH Positive Artifacts and Gas-Particle Distribution

D_4_TOH was identified by GC-MS using electron ionization - by three characteristic ions—*m*/*z* 341 (fragment ion, C_7_H_21_O_6_Si_5_^+^), 325 (fragment ion), and 343 (an isotope peak of *m*/*z* 341).^25,27,31^ Mass spectra of D_4_TOH in both gas- and particle-phase samples (Figure S3) demonstrated similar relative ion abundance to a published reference spectrum.^25,31^ D_4_TOH was detected in all four of the PUF field blanks, and were estimated by semiquantification to range 80–110 pg μL^–1^. The average value (±standard deviation) 99 (±12) pg μL^–1^ was subtracted from the concentration of D_4_TOH in all PUF samples. All reported gas-phase concentrations were above the detection limit, which was determined as the average field blank concentration plus three times their standard deviation. D_4_TOH was detected on neither QFF_f_ nor QFF_b_ field blanks. The average response area of D_4_TOH in QFF samples was 460× higher than the average response area of noise peaks in the field blank at the same retention time, so the lack of field blank subtraction is expected to have a negligible impact on the estimated semiquantified values.

D_4_TOH was observed on QFF_f_, QFF_b_, and PUF during each sampling period. Over the studied period, temperature ranged from 23.8 °C–29.2 °C, with an average of 26.4 °C (Figure S4). There were 7 of 12 sampling periods that had an equal or higher concentration of D_4_TOH on QFF_b_ than QFF_f_, which indicated that the entirety of the D_4_TOH signal from on the QFF_f_ resulted from positive sampling artifacts (Figure 3). Samples for which the D_4_TOH positive artifact was determined to be 100% occurred over a wide range of OC concentrations, and there was no statistically significant correlation between the magnitude of the D_4_TOH artifact and OC on the filter, which indicated that gas adsorption is dominated by the filter substrate and not PM on the filter. In 5 samples, the D_4_TOH concentration was higher in QFF_f_ compared to QFF_b_ (Figure 3), yet the positive artifact remained upward of 50%. Across all 12 sampling periods, the positive artifact ranged from 54 to 100%. The days with appreciable particle-phase D_4_TOH (and artifacts <100%) also had relative humidities lower than 60%; low RH has previously been demonstrated to increase particle-phase fractions of D_4_TOH adsorbing to dust by reducing competitive sorption by water.^26^ These results indicate that QFF used in this study are highly susceptible to positive artifacts and that filter-based measurements of siloxane oxidation products in PM have potential to be biased high. Correction for this positive artifact was used to obtain accurate estimates of gas-particle distributions.

**Figure 3:**
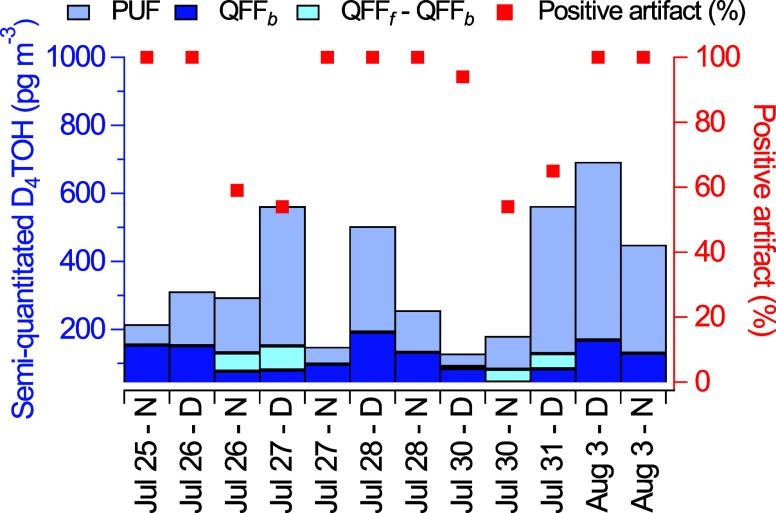
D_4_TOH concentrations determined by semiquantification in PM_2.5_ (QFF_f_ – QFF_b_), QFF-adsorbed gases (QFF_b_), and in the gas phase (PUF) during select 12 h sampling periods. The extent of adsorbed D_4_TOH on QFF, relative to the total collected on QFF is shown by red squares.

Gas-phase concentrations of D_4_TOH were calculated as the sum of the PUF and QFF_b_ for each day and ranged from 120–690 pg m^–3^, with an average of 340 pg m^–3^ (Figure 3). After artifact-correction, D_4_TOH was found to have significant particle-phase concentrations in 5 of 12 sampling periods, which ranged 6–70 pg m^–3^ and an average particle fraction of 13%. Concentrations of D_4_TOH in the gas-phase exceeded particle-phase concentrations in every sampling period (Figure 3).

As a means of method intercomparison, and validation of determination of gas-particle distributions by UPLC-MS/MS, a set of QFF_f_ samples (*n* = 7) from New York City was analyzed for D_4_TOH using both GC-MS and UPLC-MS. A strong correlation (Pearson *r* > 0.89) was observed between the two methods (Figure S5), confirming the intercomparability of the two methods and reliability of the UPLC-MS/MS method. Differences in absolute concentrations arise in part from differences in GC-MS and LC-MS semiquantification methods and estimated concentrations are expected to correlate but not match. While response factors differ across the two methods, each instrument’s response factor proportionally affects the gas and particle-phase measurements and is factored out of the gas-particle distribution calculations. Thus, there is no impact of response factor differences in the reported gas-particle distributions. On average, the LC-MS method detected 11% D_4_TOH in the particle-phase (*n* = 4), whereas the GC-MS method detected 13% in the particle-phase. For the one sample in which both methods detected D_4_TOH in the particle-phase after artifact-correction, the LC-MS method reported 9% and the GC-MS method reported 18%. Overall, the LC-MS showed a greater sensitivity to D_5_ oxidation products than the GC-MS method, while both approaches yield consistent findings, with more than 80% of D_4_TOH in the gas phase.

### D_4_TOH Gas-Particle Partition Coefficients

Although the one-month field study had a limited dynamic range in ambient temperatures (23.8–30.0 °C), there was enough dynamic range to investigate the temperature dependence of partitioning. Figure 4 shows the relationship from NYC measurements, graphed together with experimental laboratory partitioning data.^26^ The expected linear relationship between log *K*_p_ and *T* ^–1^ is seen for ambient NYC data, as expected from theory and previously demonstrated for D_4_TOH in the laboratory with model aerosol types.^26^ Lower temperatures increased the partitioning of D_4_TOH to particles and vice versa.^26^ The values for NYC ambient air fell in the middle of the range of values previously observed for diesel and woodsmoke, and were similar to those for dust, which is expected for a complex ambient aerosol mixture. Four of the five data points (shown as filled circles in Figure 4) aligned with a correlation coefficient of *r*: 0.999 and a regression equation of log *K*_p_ = (9.0 ± 0.3) *T* ^–1^ – 32.2 (±0.9) with *T* in units of K. The slope of this line for NYC ambient air was most similar to diesel particulate (8.9).^26^ One NYC ambient sample (shown as an open circle in Figure 4) had a higher *K*_p_ value than expected for its temperature, which is expected to result from TSP composition that more effectively sorbs D_4_TOH, perhaps by containing a higher dust fraction.

**Figure 4:**
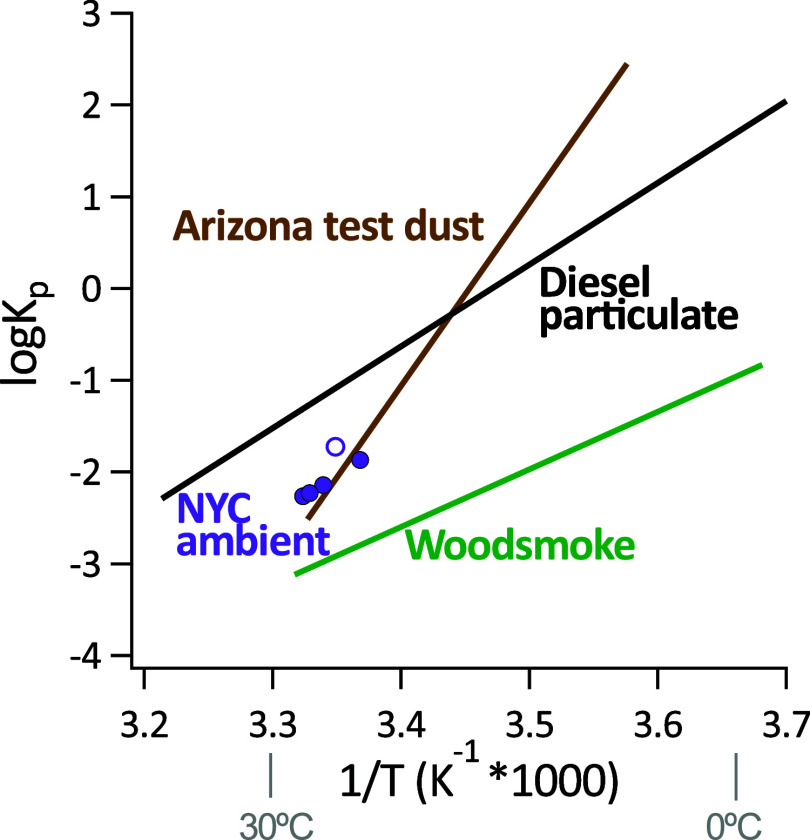
log *K*_p_ vs 1/*T* for D_4_TOH in NYC ambient aerosol and 3 particle types studied by Latimer et al. 1998.^26^ For NYC measurements, closed circles are used in linear regression, while the open circle is expected to have different particle composition.

In this study, no significant trend between *K*_p_ and relative humidity (RH) was observed. This is consistent with temperature being a major determinant in *K*_p_ and the complex nature of the ambient aerosol in NYC which contains varying mixtures of dust and combustion aerosol,^49–51^ for which RH has been shown to decrease and increase partitioning to the particle phase, respectively.

The gas-particle distributions and *K*_p_ values observed for D_4_TOH in NYC during summertime are expected to represent an annual minimum in D_4_TOH particle phase fractions, with higher particle fractions expected in winter. Following the observed linear relationship in Figure 4 for NYC ambient aerosol, it is predicted that for a TSP concentration of 10 μg m^–3^ (corresponding to the wintertime average for NYC in January, February, and December 2022 ^47^), equal concentrations of D_4_TOH in the gas and particle-phases (where *C*_p_/*C*_g_ is equal to one) would occur at 15.5 °C. For the wintertime average temperature of 2 °C in NYC,^47^ the particle-phase concentration of D_4_TOH is expected to be 34 times higher than the gas phase. Future studies should experimentally determine the gas-particle distribution and *K*_p_ under more diverse environmental conditions, especially in wintertime.

### Comparison of D_4_TOH Positive Artifacts and Gas-Particle Distributions to PAHs

Polycyclic aromatic hydrocarbons (PAH) were measured in NYC in parallel to D_4_TOH, because they cover a wide range of volatilities encompassing that of D_4_TOH and their gas-particle partitioning has been widely measured and is well understood. Ambient concentrations of PAH determined by GC-MS were artifact-corrected and their gas-particle distributions were determined in parallel to D_4_TOH (Figure S6). Semivolatile PAH with a similar vapor pressure to D_4_TOH (Table S1) demonstrate positive sampling artifacts on QFF in approximately two-thirds of samples, with average positive artifacts of 35% for phenanthrene, 73% for anthracene, and 6% for pyrene (Figure 6). However, the extent of the positive artifact is considerably higher for D_4_TOH at an average of 86% and its frequency of detection was 100% (12 of 12 samples). Other PAH found predominantly in the gas-phase (acenaphthene, naphthalene) or particle-phase (benzo­(e)­pyrene, picene) experienced negligible or undetectable artifacts on QFF. In comparison to PAH and OC (Figure 5), D_4_TOH had more frequent and a greater extent of positive artifacts on QFF, indicating it is more prone to artifact formation. The positive artifact for D_4_TOH on QFF seen in this study is likely caused by significant adsorption of D_4_TOH to the filter media. Adsorption has previously been show to be an important factor in partitioning to dust particles,^25,26^ and the same mechanism is likely active in the D_4_TOH-QFF interaction due to the silica in the QFF.

**Figure 5:**
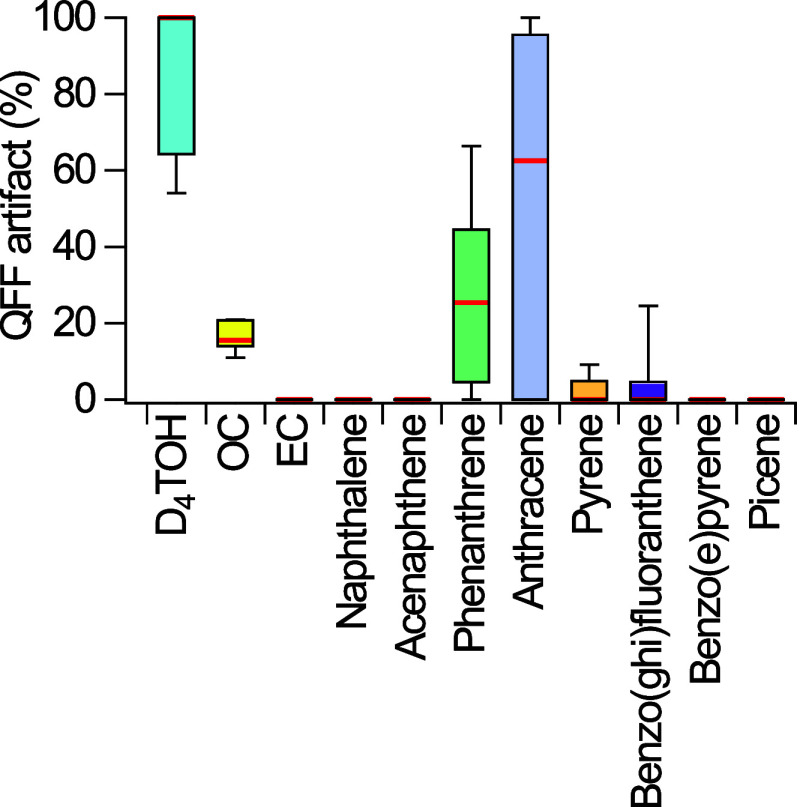
Observed positive artifacts, calculated via eq 1 for D_4_TOH, organic carbon (OC), elemental carbon (EC), and select PAH measured during NYC-METS. Data is shown as a box and whisker plot where the whiskers represent the range, the box indicates the interquartile range, and the red line represents the median.

The PAH selected for comparison contain two to seven aromatic rings and span gaseous, semivolatile, and particle-phase compounds. Particle-phase fractions in New York City (*n* = 13) for seven PAH (Figure 6) averaged to be ∼0% for naphthalene, acenaphthene, and phenanthrene, 4% for pyrene, 22% for benzo­(ghi)­fluoranthene, 99% for benzo­(e)­pyrene, and 100% for picene. Even though D_4_TOH, phenanthrene, and anthracene all experience positive artifacts, when comparing the fraction in the particle phase, D_4_TOH has a higher fraction in the particle phase despite its higher vapor pressure.

**Figure 6:**
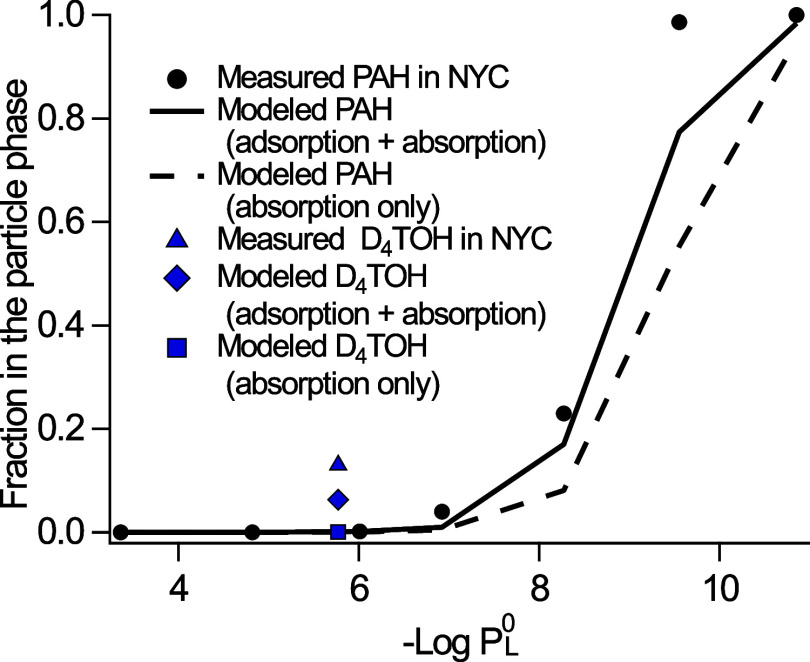
Average artifact-correct particle fractions of D_4_TOH and PAH listed in the text measured in NYC, compared to model predictions of absorption and adsorption at the average temperature of the sampling period (299.5 K).

The Pankow absorptive and adsorptive partitioning model^38^ (eq S1) was used to predict the average partitioning coefficients (*K*_p_) and distribution of D_4_TOH and PAH between gas and particle-phases in NYC ambient air. Model results show the expected trend for PAH, with higher particle fractions observed for compounds with lower vapor pressures. In a log–log plot of the compound vapor pressure versus measured *K*_p_, the slope of the line was near to the expected value of −1,^38^ at −1.08 (±0.09, Figure S7). Modeled total PAH particle fractions due to adsorption plus absorption strongly correlated with average experimental observations (Pearson’s *r*: 0.990). Contributions of adsorption and absorption to modeled particle fractions of PAH were similar, with a larger role of absorption for low vapor pressure compounds and vice versa (Table S1). In the case of D_4_TOH, adsorption leads to an estimated particle fraction of 0.06, while absorption is negligible (4 × 10^–4^). The partitioning model underpredicts the experimentally determined D_4_TOH particle fraction of 0.13 on days when particle concentrations exceeded the positive artifact by approximately a factor of 2 (Figure 6). Differences between measured and modeled values (Figure 6) are expected to arise from our assumption of unity activity coefficients; estimates of particle surface area, number of sorption sites, and enthalpy of vaporization; and the assumption of monolayer coverage. The larger role of adsorption of D_4_TOH compared to PAH originates from the difference in enthalpy of vaporization and enthalpy of desorption, which represents the strength of an interaction between an adsorbate and the adsorbing surface. In this case, D_4_TOH has a stronger interaction with the particle surface than PAH, which increases the D_4_TOH adsorptive partitioning.

### Gas-Particle Distribution of Di and Tetrasiloxanols

Successive gas-phase OH oxidation of D_4_TOH is expected to generate a series of siloxanol compounds with increasing numbers of OH groups, which were detected by UPLC-MS/MS (Figure 6). Like D_4_TOH, the gas and particle fractions were corrected for positive artifacts resulting from adsorption of gas-phase siloxanols onto the backup filter. The disiloxanol exhibited a greater tendency to partition into the particle-phase compared to D_4_TOH (discussed previously), with a corresponding particle-phase fraction of 77% (σ ± 5, Figure 7). Tetrasiloxanol was consistently detected in all particle-phase samples but was not detected in PUF samples. Normalized peak area ratios, corrected for extraction and air sample volumes, indicated that >99% of the tetrasiloxanol resided in the particle phase. The trisiloxanol was not quantifiable in either filter or PUF samples, but it is expected to predominantly partition to the particle phase, with particle fractions between those of the disiloxanol and tetrasiloxanol. Based on UPLC-MS/MS measurements (Figure S8), the positive artifact was estimated to be 80% (σ ± 16) for D_4_TOH and 10% (σ ± 4) for disiloxanol, while tetrasiloxanol was undetectable in the backup filters, indicating that positive artifacts decrease with progressive oxidation and decreasing volatility. These findings indicate that the conversion of silylmethyl to siloxanol functional groups enhances partitioning into the particle-phase. The diminishing positive artifacts with increasing oxidation further indicate that polysiloxanol oxidation products serve as more reliable tracers of SOA from D_5_ in the ambient atmosphere, because of their higher particle-phase fractions and their lesser sampling artifact in the summertime. Because the di and tetrasiloxanol measurements were applied to only four samples collected in an urban environment in the summertime, these trends should be further examined in future studies over a wider range of environmental conditions. Particle-phase products are expected to be more stable than their gas-phase products, but their stability in the ambient atmosphere remains unknown and should be the focus of further study.

**Figure 7:**
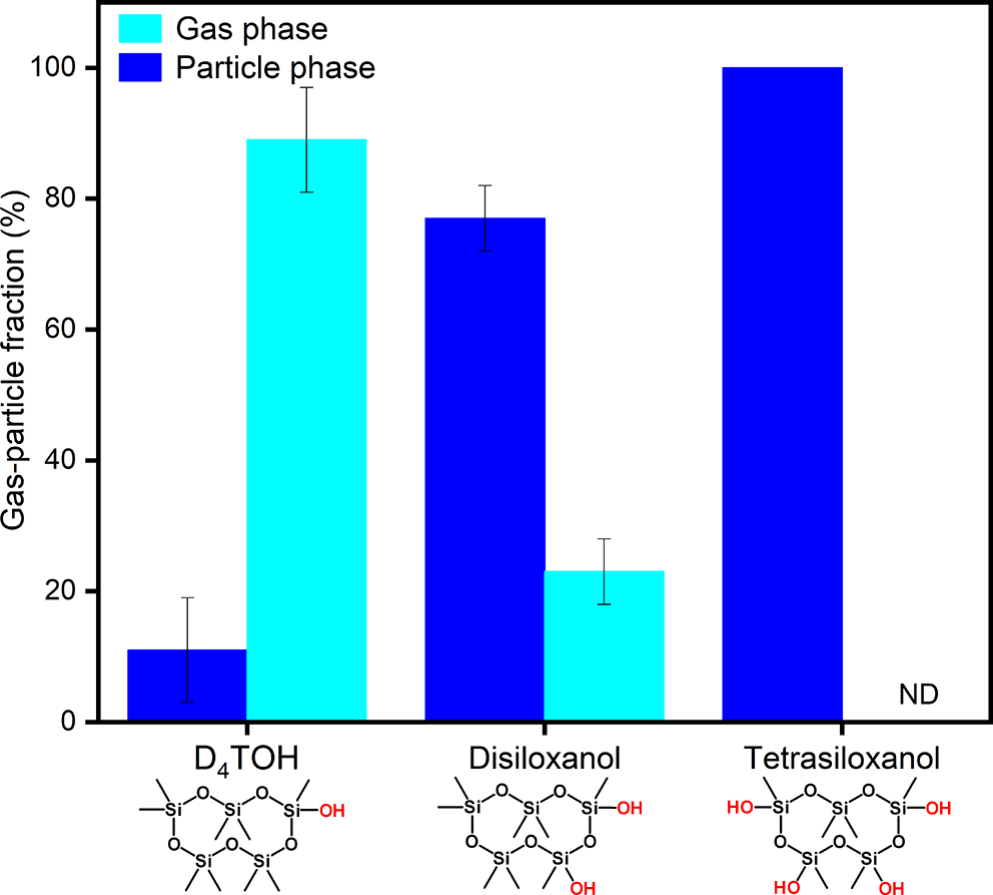
Average fractions of D_4_TOH, disiloxanol, and tetrasiloxanol detected in day and night samples collected on July 26 and 28, 2022 (*n* = 4). Trisiloxanol concentrations were below the limit of quantification.

### Implications for Tracking D_5_-Derived SOA

The detection of D_4_TOH and polysiloxanols in PM_2.5_ demonstrates that SOA derived from D_5_ contributes to ambient aerosols in New York City during summertime. D_5_ oxidation products were assessed semiquantitatively, enabling assessment of positive artifacts and gas-particle distributions, but precluding determination of their absolute concentrations. Positive artifact correction is essential when measuring D_4_TOH on QFF, as artifacts can account for the majority of the QFF signal. Alternatively, different filter media that is less prone to gas adsorption may reduce this artifact. In the case of the di and tetrasiloxanols, positive artifacts represented a small to negligible fraction of the measured PM_2.5_ value, enabling their measurement without artifact correction.

In New York City during summertime, the majority of D_4_TOH was observed in the gas-phase. D_4_TOH is expected to be reactive and further oxidize to other oxidation products, including polysiloxanols, that can increasingly partition to the particle phase.^8,27,28,52^ D_4_TOH is thus expected to be a precursor to D_5_-derived SOA and a contributor to SOA itself on some days. The majority of D_4_TOH observed in the particle phase is attributed to adsorption by the Junge-Pankow adsorptive partitioning model, with negligible contributions from absorption. Comparison to PAH, for which observed gas-particle distributions generally follow the expected trends, indicates that D_4_TOH has a uniquely high particle fraction for its vapor pressure, which is attributed to its strong interactions with the aerosol surface.

While our prior work recommended that D_4_TOH and disiloxanols may be useful tracers of SOA, based on their detectability and source specificity,^8^ the newly determined gas-particle distributions indicate that D_4_TOH is primarily in the gas phase in NYC during summertime. Disiloxanols and more oxidized products (including the tetrasiloxanol) may be more useful tracers of D_5_-derived SOA in urban aerosol during the summertime, because they are predominantly in the particle-phase. These polysiloxanol oxidation products may prove useful as molecular tracers for D_5_-derived SOA that advance source apportionment of ambient particulate matter by improving representation of anthropogenic SOA. Such source apportionment could be achieved by chemical mass balance modeling through the development of SOA tracer to OC ratios in controlled chamber studies, employing the SOA-tracer approach.^53^ Alternatively, these candidate tracers could be incorporated into positive matrix factorization (PMF) to potentially resolve a D_5_-derived SOA source through multivariate modeling. Either of these approaches may be used to gain insight into the relative importance of D_5_-derived SOA compared to other primary and secondary sources.

## Supplementary Material



## Notes

Pankow absorptive and adsorptive partitioning model equations and term definitions (Equation S1); select thermodynamic values for PAH and D_4_TOH (Table S1); Spike recovery data for PAH (Figure S1); organic and elemental carbon concentrations in PM_2.5_ (Figure S2); mass spectral, chromatographic, and isotopic distribution information for D_4_TOH (Figure S3); relative humidity and temperature data (Figure S4); GC-MS and UPLC-MS/MS method intercomparison (Figure S5); detailed PAH gas-particle concentration data and positive artifact data (Figure S6); log–log plot of *P*_L_^0^ versus *K*_p_ for PAH and D_4_TOH (Figure S7); and polysiloxanol positive artifact data (Figure S8) (PDF)
