# Supplementary material for: Gas-Particle Distribution of D5 Oxidation Products in New York City during Summertime
Source: ACS EST Air. 2025 Oct 16;2(11):2517–26. doi: 10.1021/acsestair.5c00193 (PMC12624521; doi:10.1021/acsestair.5c00193)
Supplement: Supplementary file 1 [file ea5c00193_si_001.pdf]

Supporting Information for:

Gas-particle distribution of D_5 oxidation products in New York City during summertime

Authors: Josie K. Welker[1], Jeewani N. Meepage[1], Charles O. Stanier[2], Elizabeth A. Stone*[1,2]

[1]University of Iowa, Department of Chemistry, Iowa City, Iowa 52242

[2]University of Iowa, Department of Chemical and Biochemical Engineering, Iowa City, Iowa 52242

This file includes:

Number of pages: 10

Number of figures: 8

Number of tables: 1

Number of equations: 1

Pankow Absorption and Junge-Pankow Adsorption Model Gas-Particle Partitioning Equation

The partitioning coefficient, K_p ($m^3\ \mu g^{-1}$), was estimated for PAH and D_4TOH using Equation S1 from Pankow 1994 with consideration of adsorptive and absorptive partitioning.[1]

$$K_p = \frac{1}{P_L^0}\left[N_s A_{TSP} RT e^{(Q_l - Q_v/RT)} + \frac{f_{om} RT}{\varepsilon MW_{om} 10^6}\right] \quad (S1)$$

The sub-cooled vapor pressure (P^0_L atm) was obtained from Estimation Program Interface suite™ version 4.11 from the Environmental Protection Agency (EPA)[2] for PAH and Jang et al. 1997[3] for D_4TOH. The Clausius-Clapeyron equation was used to relate the change in vapor pressure of PAH and D_4TOH with the average temperature during the sampling periods (299.5 K).

The first term within the brackets of Equation S1 represents the contributions from adsorptive partitioning. The moles of sorption sites on aerosol (N_s, mol cm^{-2}) is calculated as the inverse of Avogadro's number and surface area per sorption site (cm^2). For the surface area per sorption site, the value 1.6×10^{-15} cm^2 was based upon Langmuir 1918[4], which defines the surface area of N_2 gas molecules in molecules cm^{-2} in Langmuir monolayer adsorption model. The surface area of total suspended particles (A_{TSP}) was calculated as 0.032 $cm^2\ \mu g^{-1}$ for 13 $\mu g\ m^{-3}$ TSP value in a study applying the Junge-Pankow model.[5] This value was used for A_{TSP}, as the TSP of 13 $\mu g\ m^{-3}$ is close to our estimated NYC TSP concentration of 17 $\mu g\ m^{-3}$ and changes in TSP values of less than one order of magnitude are not expected to have a large effect on A_{TSP}.[5] The ideal gas constant (R) was expressed as 8.2057×10^{-5} m^3 atm mol^{-1} K^{-1}. The average temperature (T) over the sampling period in NYC was 299.5 K. The enthalpy of desorption (Q_l, kJ mol^{-1}) used for D_4TOH was 80 kJ mol^{-1}, based on estimates for chemisorption or organic compounds on to inorganic surfaces.[6] For the PAH, Q_l was calculated using the equation $Q_l = 0.88\ Q_v + 16.56$ ($r^2 = 0.991$) from Pankow et al. 1991.[7] The enthalpy of vaporization (Q_v) for PAH were sourced from the literature[8] and for D_4TOH was sourced from a prediction by ChemSpider (RSC) generated using ACD/Labs PhysChem Module version 14.00.[9]

The second term within the brackets of Equation S1 represents contributions from absorptive partitioning. The weight fraction of absorbing organic matter (f_{om}) phase (0.37) was calculated as the average organic matter (om) concentration divided by the average TSP concentration. The average om concentration was calculated as the average OC concentration multiplied by 1.72. The mean molecular weight of the absorbing organic material (MW_{om},) was set to 250 g mol^{-1}, which was previously modeled as an estimate for organic matter in urban areas.[10] This value is also within the range of 100-300 g mol^{-1} recommended by Pankow et al. 1994.[1] The activity coefficient ε accounts for non-ideal behavior in the aerosol phase; a value of 1 was used, which is at the lower end of the range recommended by Pankow et al. 1994 of (1-5).[1] The value 10^6 is a unit conversion factor ($\mu g\ g^{-1}$).

Table S1: Parameters used to calculate gas-particle partitioning of the selected PAHs and D_4TOH.

Compound	Number of carbon atoms	Molecular mass (g mol^{-1})	P_L^0 at 298.15 K (atm)[2]	Enthalpy of vaporization (kJ mol^{-1})[8, 9]	Enthalpy of desorption (kJ mol^{-1})	Partitioning coefficient (K_p) (m^3 μg^{-1})	Contribution from adsorption	Contribution from absorption
**Naphthalene**	10	128.17	3.93×10^{-4}	43.2	54.6	2.64×10^{-7}	68%	32%
**Acenaphthene**	12	154.21	1.34×10^{-5}	51.1	61.5	5.96×10^{-6}	59%	41%
**Phenanthrene**	14	178.24	8.63×10^{-7}	52.7	62.9	8.82×10^{-5}	58%	42%
**Anthracene**	14	178.24	6.50×10^{-7}	52.4	62.7	1.18×10^{-4}	58%	42%
**Fluoranthene**	16	202.26	7.99×10^{-8}	60.2	69.5	7.74×10^{-4}	49%	51%
**Pyrene**	16	202.25	1.05×10^{-7}	61	70.2	5.78×10^{-4}	48%	52%
**Benzo(ghi)fluoranthene**	18	226.28	4.60×10^{-9}	64.7	73.5	1.20×10^{-2}	43%	57%
**Benzo(e)pyrene**	20	252.32	2.42×10^{-10}	70.8	78.9	2.01×10^{-1}	36%	64%
**Picene**	22	278.33	1.19×10^{-11}	75.7	83.2	3.74	31%	69%
**D_4TOH**	9	372.75	9.13×10^{-7}	57.5	80	3.95×10^{-3}	99%	1%

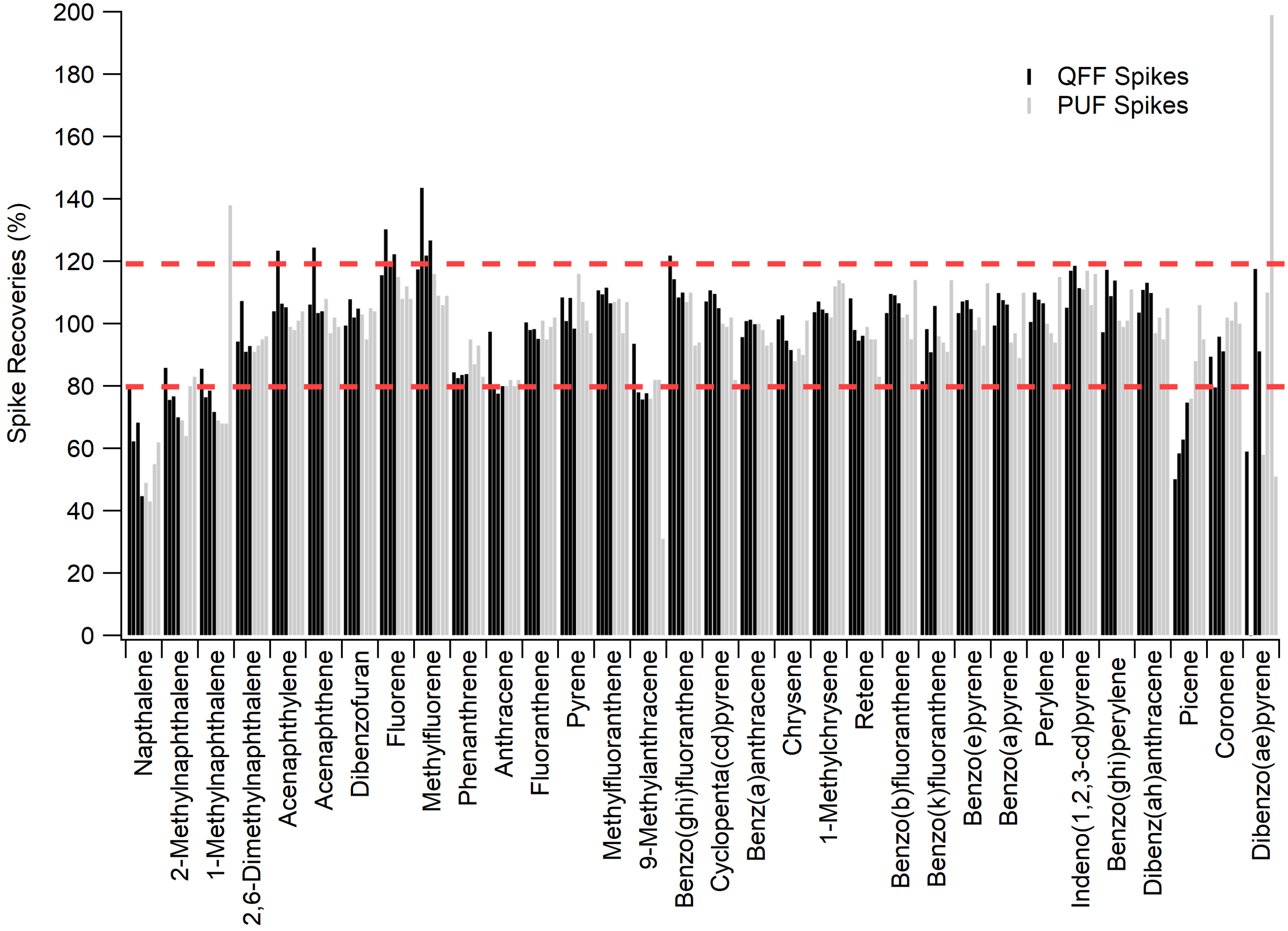

Figure S1. Spike recovery percentage for polycyclic aromatic hydrocarbons, with QFF (n=4) shown in black and PUF (n=4) shown in gray. Red dashed lines mark the acceptable range of recovery (80-120%)

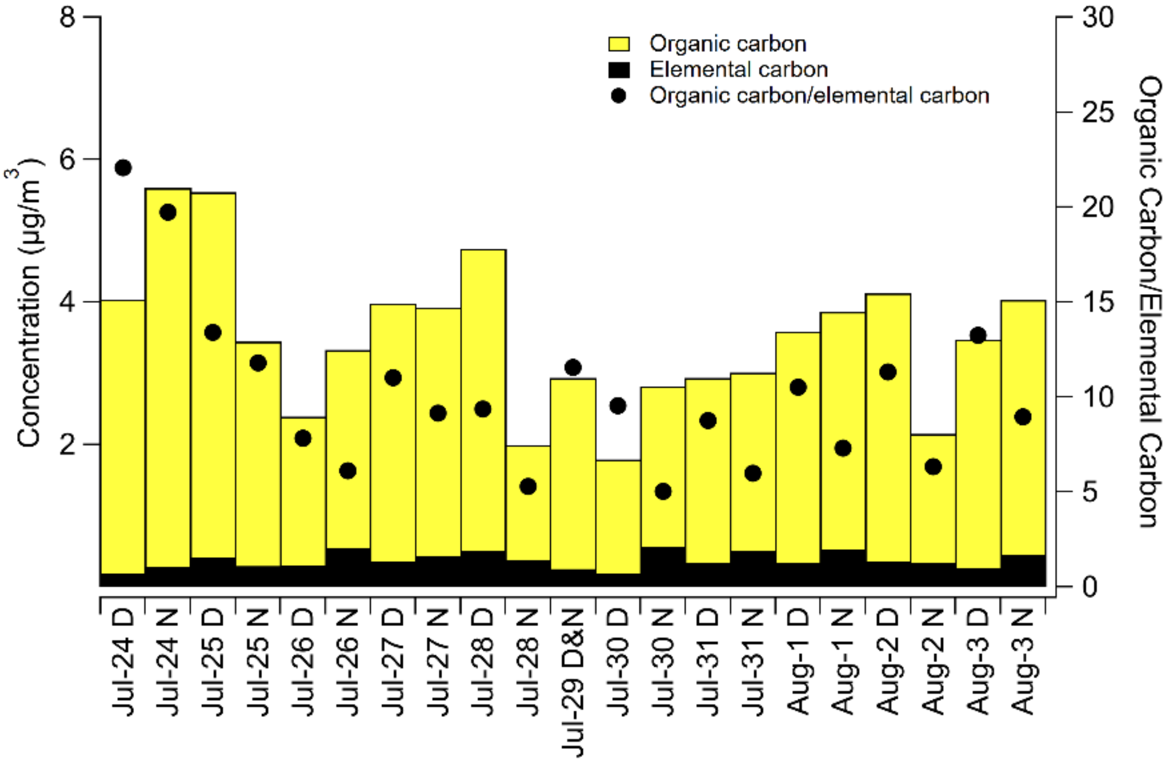

Figure S2. Organic and elemental carbon concentrations in $PM_{2.5}$, with organic carbon to elemental carbon ratios denoted by black circles.

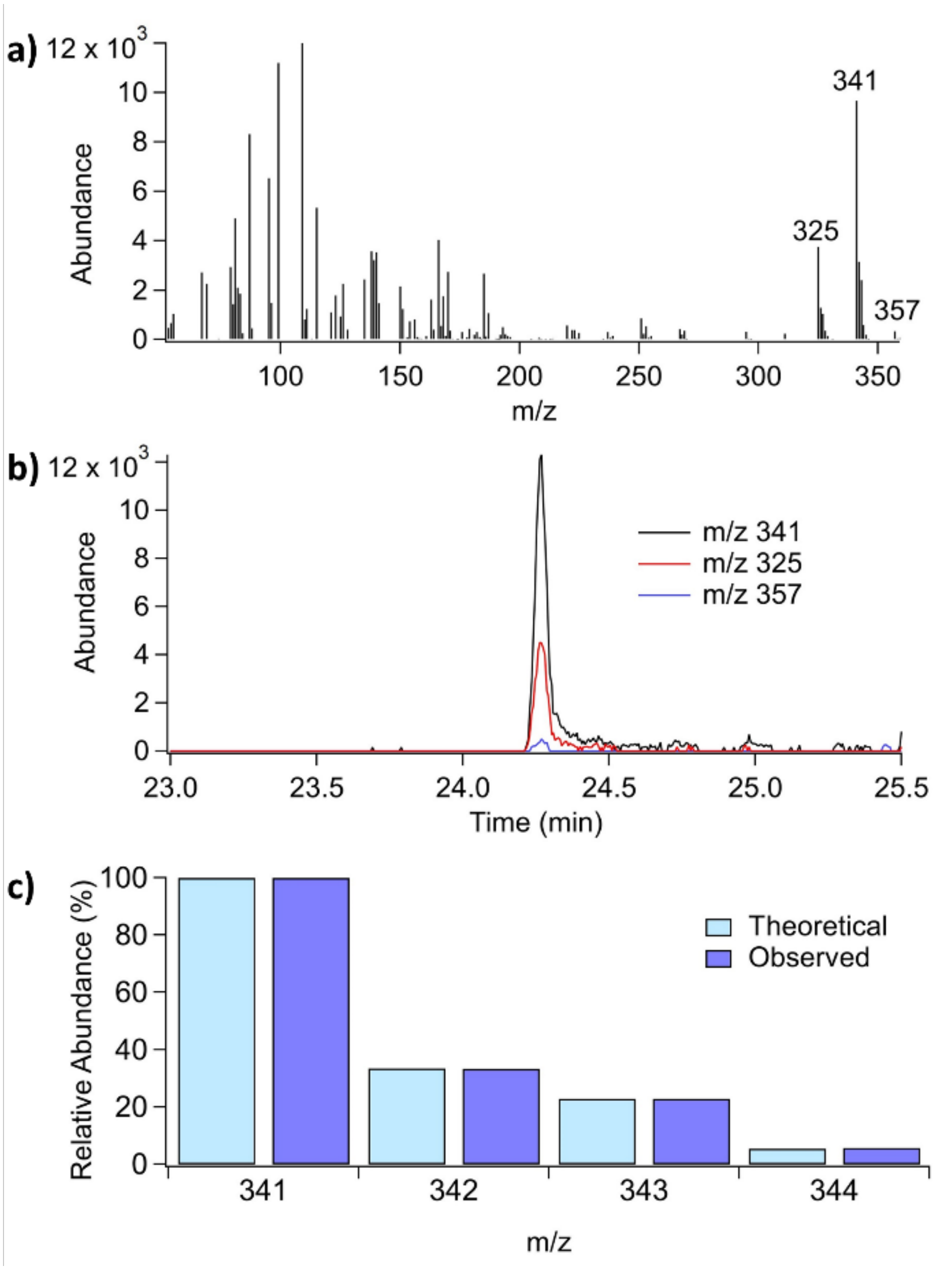

Figure S3. a) Background-subtracted mass spectrum of D_4TOH. b) Selected ion chromatogram of D_4TOH. c) Observed and theoretical isotope distribution for $C_7H_{21}O_6Si_5^+$

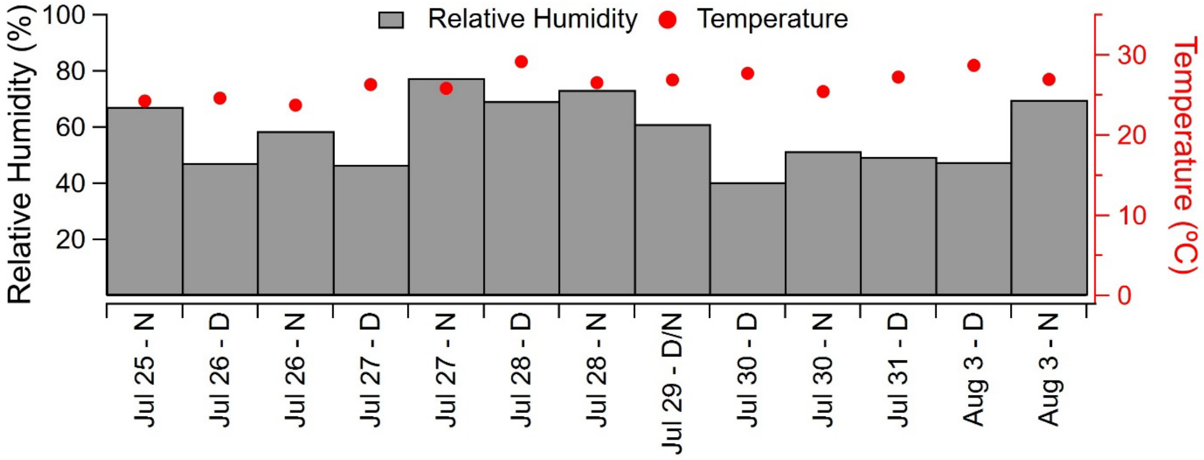

Figure S4. Average relative humidity (%)and temperature (°C) for sampling periods when QFF_b were collected.

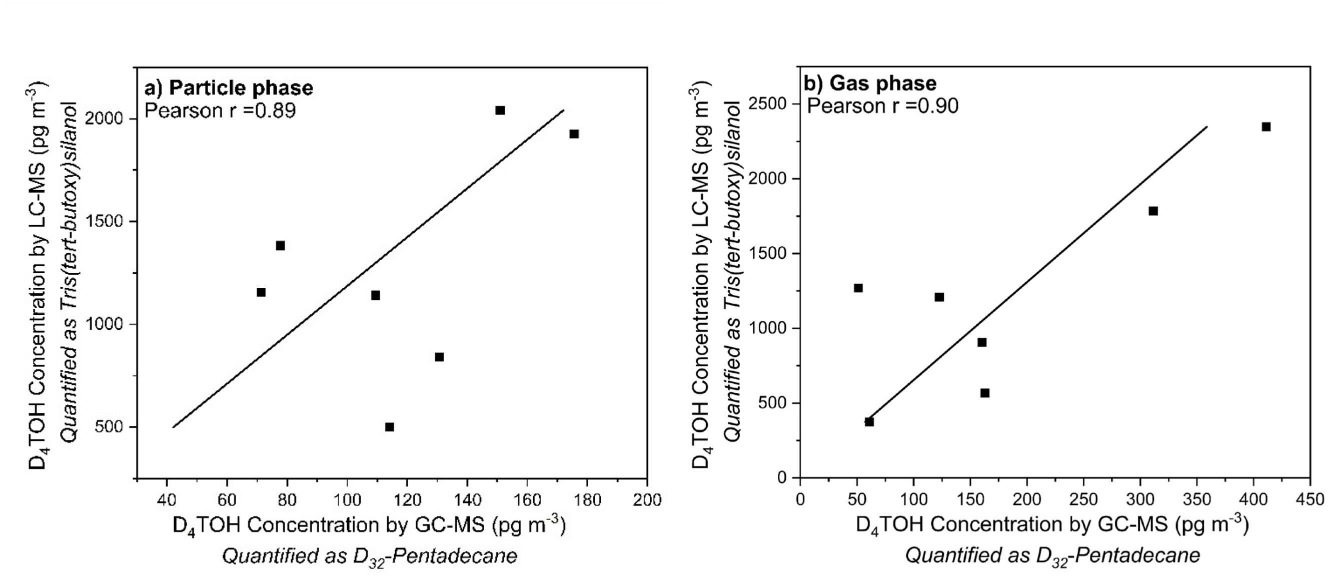

Figure S5. (a) Particle-phase and (b) gas-phase D_4TOH concentrations measured using GC-MS and UPLC-MS/MS for seven NYC-METS field site samples.

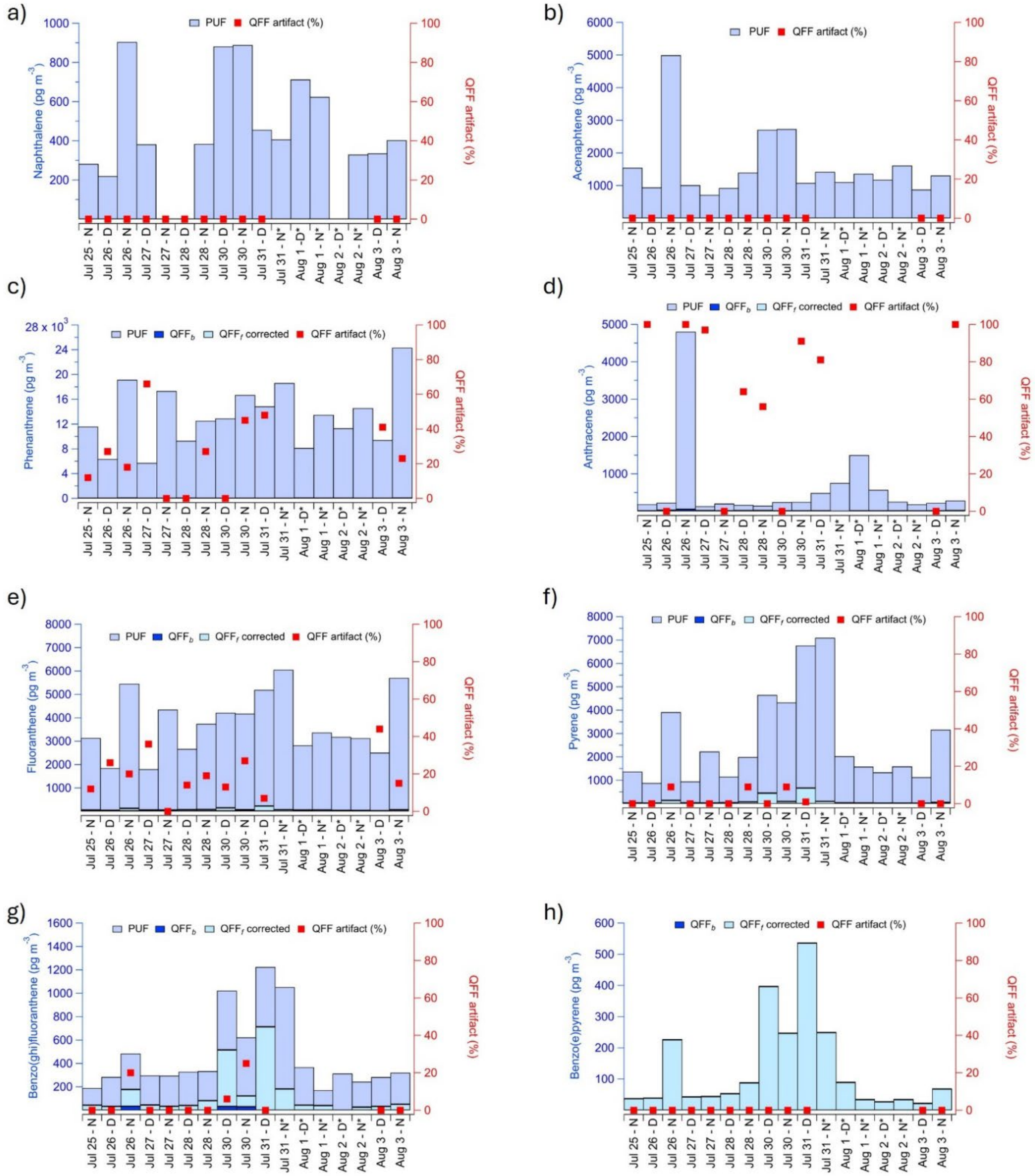

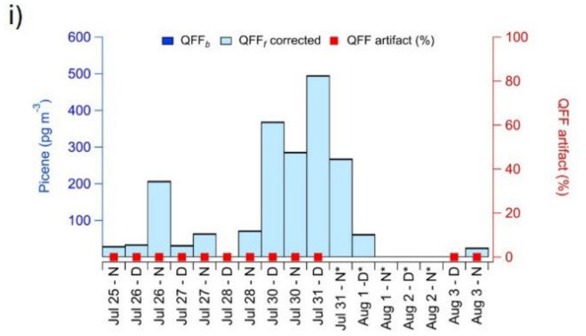

Figure S6. The quantification of PAHs distributed among the particle phase (QFF$_f$) and gas phase (QFF$_b$ and PUF), with the positive gas phase artifact represented by red square markers. On days denoted with an asterisk (*) there is no QFF$_b$ data available, and no QFF artifact measured.

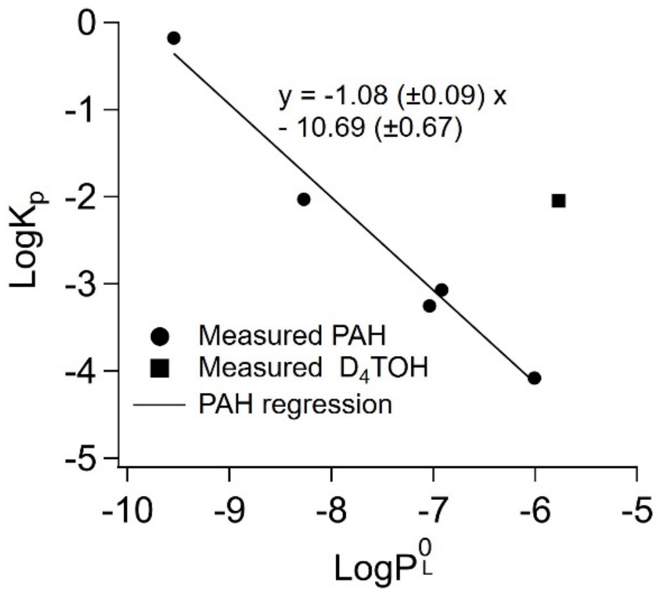

Figure S7. A log-log plot of K_p (m^3 μg^{-1}) and P^0_L (atm) for average measured PAH and D_4TOH for the nighttime sample that began August 3.

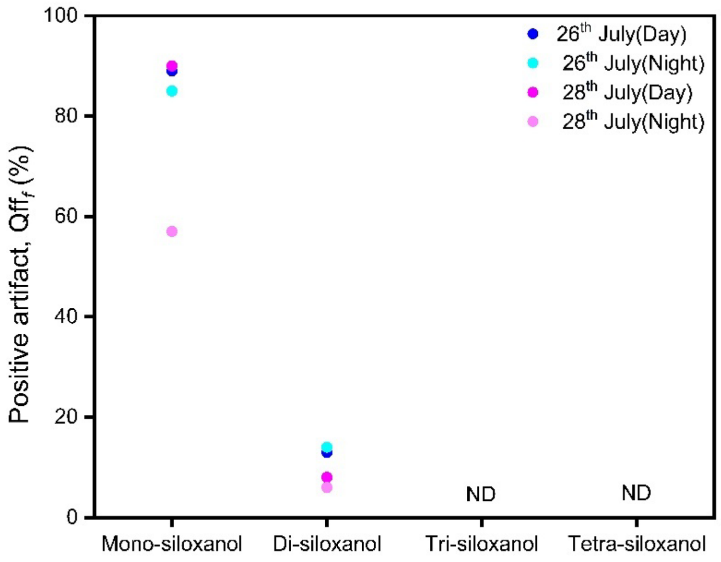

Figure S8: Positive artifacts on QFF_f (%) attributed to multistep oxidation products in the four tested samples, with darker shades representing day samples and lighter shades representing night samples.

References

1. Pankow, J. F., An Absorption-Model of Gas-Particle Partitioning of Organic-Compounds in the Atmosphere. *Atmospheric Environment* **1994,** *28* (2), 185-188.

2. EPA, U. *Estimation Programs Interface Suite™ for Microsoft® Windows*, 4.11; United States Environmental Protection Agency: Washington, DC, USA, 2012.

3. Jang, M.; Kamens, R. M.; Leach, K. B.; Strommen, M. R., A Thermodynamic Approach Using Group Contribution Methods to Model the Partitioning of Semivolatile Organic Compounds on Atmospheric Particulate Matter. *Environmental Science & Technology* **1997,** *31* (10), 2805-2811.

4. Langmuir, I., THE ADSORPTION OF GASES ON PLANE SURFACES OF GLASS, MICA AND PLATINUM. *J Am Chem Soc* **1918,** *40* (9), 1361-1403.

5. Salthammer, T.; Schripp, T., Application of the Junge- and Pankow-equation for estimating indoor gas/particle distribution and exposure to SVOCs. *Atmospheric Environment* **2015,** *106*, 467-476.

6. Chandramouli, B.; Kamens, R. M., The photochemical formation and gas-particle partitioning of oxidation products of decamethyl cyclopentasiloxane and decamethyl tetrasiloxane in the atmosphere. *Atmospheric Environment* **2001,** *35*, 87-95.

7. Pankow, J. F., Common y-intercept and single compound regressions of gas-particle partitioning data vs 1/T. *Atmospheric Environment. Part A. General Topics* **1991,** *25* (10), 2229-2239.

8. Karcher, W.; Commission of the European Communities. Joint Research Centre. Petten Establishment., *Spectral atlas of polycyclic aromatic compounds : including data on occurrence and biological activity*. D. Reidel Pub. Co. for the Commission of the European Communities; Sold and distributed in the U.S.A. and Canada by Kluwer Academic Publishers: Dordrecht ; Boston Hingham, MA, 1985.

9. CSID:111127535. https://www.chemspider.com/Chemical-Structure.111127535.html.

10. Yazdani, A.; Dillner, A. M.; Takahama, S., Estimating mean molecular weight, carbon number, and OM/OC with mid-infrared spectroscopy in organic particulate matter samples from a monitoring network. *Atmos. Meas. Tech.* **2021,** *14* (7), 4805-4827.
